# Sulforaphane Protects Pancreatic Acinar Cell Injury by Modulating Nrf2-Mediated Oxidative Stress and NLRP3 Inflammatory Pathway

**DOI:** 10.1155/2016/7864150

**Published:** 2016-10-26

**Authors:** Zhaojun Dong, Haixiao Shang, Yong Q. Chen, Li-Long Pan, Madhav Bhatia, Jia Sun

**Affiliations:** ^1^State Key Laboratory of Food Science and Technology, School of Food Science and Technology, Jiangnan University, Wuxi 214122, China; ^2^Jiangnan University School of Medicine, Wuxi 214122, China; ^3^Shanghai Key Laboratory of Bioactive Small Molecules and Department of Pharmacology, School of Pharmacy, Fudan University, Shanghai 201203, China; ^4^Inflammation Research Group, Department of Pathology, University of Otago, Christchurch, 2 Riccarton Avenue, P.O. Box 4345, Christchurch 8140, New Zealand

## Abstract

Acute pancreatitis (AP) is characterized by early activation of intra-acinar proteases followed by acinar cell death and inflammation. Cellular oxidative stress is a key mechanism underlying these pathological events. Sulforaphane (SFN) is a natural organosulfur antioxidant with undescribed effects on AP. Here we investigated modulatory effects of SFN on cellular oxidation and inflammation in AP. AP was induced by cerulean hyperstimulation in BALB/c mice. Treatment group received a single dose of 5 mg/kg SFN for 3 consecutive days before AP. We found that SFN administration attenuated pancreatic injury as evidenced by serum amylase, pancreatic edema, and myeloperoxidase, as well as by histological examination. SFN administration reverted AP-associated dysregulation of oxidative stress markers including pancreatic malondialdehyde and redox enzymes superoxide dismutase (SOD) and glutathione peroxidase (GPx). In acinar cells, SFN treatment upregulated nuclear factor erythroid 2-related factor 2 (Nrf2) expression and Nrf2-regulated redox genes including quinoneoxidoreductase-1, heme oxidase-1, SOD1, and GPx1. In addition, SFN selectively suppressed cerulein-induced activation of the nucleotide-binding domain leucine-rich repeat containing family, pyrin domain-containing 3 (NLRP3) inflammasome, in parallel with reduced nuclear factor- (NF-) *κ*B activation and modulated NF-*κ*B-responsive cytokine expression. Together, our data suggested that SFN modulates Nrf2-mediated oxidative stress and NLRP3/NF-*κ*B inflammatory pathways in acinar cells, thereby protecting against AP.

## 1. Introduction

Acute pancreatitis (AP) has long been considered as a disorder of pancreatic self-digestion, in which premature activation and retention of intracellular proteases induce acinar cell injury and death [1, 2]. Local pancreatic inflammation follows and when excessive, develops into systemic inflammatory responses with multiple organ dysfunction involved, which result in significant morbidity and mortality [3]. Although the precise mechanisms of AP remain incompletely understood, a key role of oxidative stress, inflammatory cell infiltration, and inflammatory mediators in the pathophysiology of AP has been suggested [4].

Reactive oxygen species (ROS) and resultant oxidative stress are reported to be important effectors in a wide variety of inflammatory diseases, including AP [5, 6]. Under physiological conditions, the detrimental ROS can be rapidly detoxified by endogenous enzymatic or nonenzymatic antioxidants. Antioxidant enzymes like superoxide dismutase (SOD), glutathione peroxidase (GPx), nicotinamide adenine dinucleotide phosphate (NADPH), quinoneoxidoreductase-1 (NQO1), and heme oxidase-1 (HO-1) protect cells against oxidative stress [7]. Nuclear factor erythroid 2-related factor 2 (Nrf2), a member of the nuclear factor erythroid 2 family of nuclear basic leucine zipper transcription factors, is a master regulator of these constitutively expressed cytoprotective enzymes by binding to the antioxidant responsive element (ARE) [8]. During AP, excessive production of ROS and a decreased capacity of intrinsic antioxidative defense system lead to peroxidation of lipid membranes, cytoskeleton disintegration, genetic alterations, and ultimately cell death [9, 10]. Also, oxidative stress contributes to not only the amplification of pancreatic damage but also the progression of local inflammation to systemic inflammatory responses [6]. Therefore, supplementation with exogenous antioxidants represents a beneficial therapeutic strategy or a useful adjunctive option in the treatment of AP [11, 12].

Sulforaphane (SFN) is a naturally occurring isothiocyanate, mainly present in broccoli and other cruciferous vegetables, and consumed widely as a regular dietary component [13]. SFN is known to be important in the maintenance of cellular redox balance [14] and has earlier been used as an effective phytochemical antioxidant in studies of gastrointestinal and pulmonary diseases [15–17]. In addition, SFN has been reported to inhibit multiple inflammasomes in immune cells [18]. Recent findings indicate that the nucleotide-binding domain leucine-rich repeat containing family, pyrin domain-containing 3 (NLRP3) inflammasome, provides the link between acinar cell death and inflammation during the development of AP; and activation of NLRP3 inflammasome is involved in the development of AP [19], which is inhibited by antioxidants like hydrogen-rich saline [12].

In light of recent advances, however, the therapeutic potential of SFN in AP or the mechanism thereof is unknown. In the present study, therefore, we investigated modulatory effects of SFN as a ROS scavenger and anti-inflammatory nutrient compound in cerulein-induced AP and the underlying cellular mechanisms.

## 2. Methods

### 2.1. Animals

All animal experiments were approved by the Jiangnan University Institutional Animal Ethics Committee and carried out in compliance with national and international (Declaration of Helsinki) guidelines for the Care and Use of Animal Laboratory Animals. BALB/c mice (20–25 g) were divided randomly into control or experimental groups and housed in the Animal Housing Unit of Jiangnan University under a 12-hour light/dark cycle with unlimited access to food and water. All mice were adjusted to laboratory conditions over the course of 1 week prior to the experiments.

### 2.2. Induction of AP & Pretreatment with SFN

BALB/c mice received 8 hourly intraperitoneal (i.p.) injections of 0.9% wt/vol saline or saline containing 50 *μ*g/kg body wt of cerulein (Sigma-Aldrich, St. Louis, USA), as described previously [1]. To examine the biological effects of SFN (LKT Laboratories, St. Paul, USA), mice were treated with SFN (5 mg/kg, i.p.) dissolved in 0.9% wt/vol saline once a day for 3 consecutive days before the induction of AP [1, 17, 20]. Animals were sacrificed by an i.p. injection of a lethal dose of pentobarbitone 1 hour after the final cerulein or saline injection. Samples of pancreas and blood were rapidly harvested. Quantification of pancreatic injury was performed on circulating amylase activity, pancreatic water content, pancreatic myeloperoxidase activity, and morphologic extent of acinar cell injury as described previously [21].

### 2.3. Determination of Pancreatic Water Content

The water content of the pancreas was quantified by comparing the weight of the freshly harvested samples of pancreas (wet weight) with the weight of the same samples after desiccation at 60°C for 72 hours (dry weight) [22]. The results were expressed as percentage water content.

### 2.4. Determination of Serum Amylase Activity

 Fresh blood was collected by removing eyeball. Following centrifugation, serum was removed and kept frozen at −80°C until assayed. Serum amylase activity was assessed with a commercially available kit (Nanjing Jiancheng Bioengineering Institute, Nanjing, China) based on the use of amylon as the substrate for *α*-amylase. The indine can be combined with unhydrolyzed amylon and build some blue compounds; we can calculate the AMS activity according the blue compounds from hydrolyzed amylon. The soluble chromogen in 0.1 mL of serum was measured spectrophotometrically at 660 nm. The absorbance was linear with enzyme activity following the manufacturer's instructions.

### 2.5. Quantification of Pancreatic Myeloperoxidase (MPO) Activity

Sequestration of neutrophils within the pancreas was evaluated by measuring pancreas MPO activity [23]. The MPO assay kit was purchased from Nanjing Jiancheng Bioengineering Institute (Nanjing, China). Pancreas samples were weighed and homogenized in 0.86% wt/vol saline on ice for determination of pancreas MPO activity and other studies. Tissue homogenates for studies of lipid peroxidation and cytokines were centrifuged at 4°C at 500 ×g for 10 min and 14,000 ×g for 15 min, respectively. Aliquots of the clear supernatants were used for each assay. MPO activity was assessed spectrophotometrically at 460 nm. The results were expressed as the activity U/g wet tissue.

### 2.6. Determination of Malondialdehyde (MDA), SOD, and GPx

MDA, a reliable marker of lipid peroxidation, was measured using the thiobarbituric acid (TBA) method, with 1,1,3,3-tetramethoxypropane as a standard [24]. Briefly, 2 mL of fresh solution with 15% w/v trichloroacetic acid (TCA), 0.375% w/v TBA, and 0.25 mL/L HCl was added to 1 mL of 10% pancreas homogenate with 1.15% KCl. The mixture was heated at 95°C for 15 min, cooled to room temperature using tap water, and centrifuged at 300 ×g for 5 min. The supernatant was collected in plastic tubes and centrifuged at 9000 ×g for 30 min. Absorption of the pink supernatant was determined spectrophotometrically at 532 nm. The amount of MDA was expressed as nmol/mg wet tissue. SOD and GPx levels in homogenized pancreas tissue were measured using commercial kits (Nanjing Jiancheng Bioengineering Institute, Nanjing, China).

### 2.7. Determination of Pancreatic IL-1*β* and TGF-*β* Protein Levels

Pancreatic IL-1*β* and TGF-*β* levels were measured with ELISA kits from Dobio Biology Technology Inc (Shanghai, China) according to the manufacturer's protocol. Samples were measured with a microplate reader Multiskan GO (Thermo Fisher Scientific Inc, Vantaa, Finland).

### 2.8. Histological Studies and Evaluation of Pancreatic Morphology

For morphological analysis, small pieces of the pancreas samples were fixed in 4% neutral paraformaldehyde, embedded in paraffin, and sectioned. The 4 *μ*m thick sections were stained with hematoxylin and eosin (H&E) for routine histology as previously described [25]. For H&E staining, sections were stained with hematoxylin for 10 min, washed, and stained with 0.5% eosin for an additional 5 min. After a washing step with water, the slides were dehydrated in 70%, 96%, and 100% ethanol and then in xylene. Pancreatic injury was examined by random selection of microscopic fields of each tissue sample and evaluated based on the presence of acinar cell degeneration, edema, and infiltration of inflammatory cells, which are markers of pancreatic damage and inflammation. Representative graphs were selected by blinded investigators unknown of the sample groups.

### 2.9. Immunohistochemistry

Immunostaining was performed on 4 *μ*m thick sections after deparaffinization as described above. Microwave antigen retrieval was performed in citrate buffer (pH 6.0) for 10 min prior to peroxidase quenching with 3% H_2_O_2_ in PBS for 10 min. Sections were then washed in water and preblocked with 5% BSA for 1 hour. In the primary antibody reaction, slides were incubated for 1 hour overnight at 4°C in a 1 : 500 dilution of antibody. The sections were then incubated with goat anti-rabbit HRP-conjugated secondary antibody for 1 hour at room temperature. Following a washing step with PBS, streptavidin-HRP was applied. Finally, the sections were developed with diaminobenzidine tetrahydrochloride substrate for 10 min and counterstained with hematoxylin. Slides were scanned by Pannoramic MIDI Digital Slide Scanner (3D-Histech Co., Ltd., Budapest, Hungary) and images were captured.

### 2.10. Preparation and Treatment of Mouse Primary Pancreatic Acinar Cells

Mouse pancreatic acinar cells were freshly isolated by collagenase digestion as described previously [26]. Briefly, pancreas from BALB/c mice (20–25 g) were removed, infused with collagenase solution (HBSS 1x containing 10 mM HEPES, 200 U/mL of collagenase, and 0.25 mg/mL of trypsin inhibitor), and incubated in a shaking water bath for 20~30 min at 37°C. The digested tissue was centrifuged and washed twice with cold buffered washing solution (HBSS 1x containing 5% FBS and 10 mM HEPES). A cell suspension in complete medium consisting of only small clumps, around 3–5 acinar cells, was used to carry out the following experiments. These pancreatic acinar cells were pretreated with SFN at a concentration of 10 *μ*M for 30 min and then stimulated with 0.5 *μ*M cerulein for 1 hour at 37°C [27, 28] after which the acinar cells were subjected to ROS assay, RNA isolation for quantitative real-time PCR analysis, and protein extraction for western blot analysis, respectively.

### 2.11. ROS Assays

After treatment with cerulein in the absence or the presence of SFN, pancreatic acinar cells were incubated with fluorescence dye DCFH-DA (Sigma-Aldrich, St. Louis, USA) in the dark for 30 min at 37°C [29]. Then, the cells were washed with PBS three times. Fluorescence absorbance was detected with excitation/emission wavelengths of 488 nm/543 nm and relative ROS level was expressed as the mean percentage of fluorescence absorbance in treated versus control cells.

### 2.12. RNA Isolation and Quantitative Real-Time PCR Analysis

For Q-PCR analysis of adhesion mRNA expression, total cellular RNA was extracted from the pancreatic acinar cells using TRIzol reagent (Invitrogen, Carlsbad, CA) following the manufacturer's instructions with some modifications. Briefly, after treatment, cells were immediately homogenized in TRIzol. Following centrifugation, phase separation was achieved with chloroform. The aqueous phase was transferred to a new microfuge tube and RNA was precipitated by adding isopropyl alcohol. After RNA was pelleted by centrifugation (12,000 ×g for 10 min at 4°C), the pellet was washed twice in 70% ethanol, air-dried, and dissolved in RNase-free water. 1 *μ*g of total RNA was reversely transcribed into cDNA by the PrimeScript™ RT reagent kit with gDNA Eraser (Takara, Tokyo, Japan), according to the manufacturers' instructions. Quantitative real-time PCR amplification was carried out using the iTaq™ Universal SYBR Green Supermix (Bio-Rad, CA, USA) and the specific primers (Table 1) were carried out on the Bio-Rad CFX Connect Real-time System (Bio-Rad, Hercules, CA). The PCR reactions were performed at 95°C for 5 min and subjected to 40 cycles of 95°C for 15 s and 60°C for 60 s. The relative levels of each target gene mRNA transcripts to the control were calculated using the comparative 2^−ΔΔCT^ method.

### 2.13. Preparation of Nuclear and Cytosolic Cell Extract or Whole-Cell Lysates for Western Blot Analysis

After treatment, pancreatic acinar cells were homogenized on ice in radio immunoprecipitation assay lysis buffer supplemented with protease inhibitor cocktail and phosphatase inhibitor cocktail (Sangon Biotech, Shanghai, China) and centrifuged at 4°C for 15 min at 14,000 rpm. The supernatants were collected as whole-cell lysates and stored at −80°C. Cell nuclear fractions and cytosolic fraction were subsequently prepared using a commercial kit (Beyotime, Shanghai, China). Protein concentrations were determined by the BCA Protein Assay Kit (Thermo Fisher Scientific).

### 2.14. Western Blotting for Nrf2, p65, and NLRP3 Inflammasome

Different extracted proteins were separated by 10% or 12% SDS-PAGE and the resolved proteins were transferred to a PVDF membrane. The membrane was blocked with 5% w/v nonfat dry milk in TBS-T (0.1% Tween 20 in TBS). The membrane was then incubated with a 1 : 500 dilution of primary antibody. Antibodies against Nrf2, p65 subunit of NF-*κ*B (NF-*κ*B p65), p20 subunit of caspase-1 (casp-1-p20), and cleaved IL-1*β* were obtained from Santa Cruz Biotechnology (CA, USA), and antibody against NLRP3 was obtained from Cell Signaling Technology (Beverly, USA). After thorough washing with TBS-T, a 1 : 2000 dilution of goat anti-rabbit or rabbit anti-goat HRP-conjugated secondary antibody in TBS-T was applied to the membrane and the blot was developed using the Pierce™ ECL Western Blotting Substrate (Thermo Fisher Scientific) before exposure using FluorChem system. Histone and tubulin (Cell Signaling, Beverly, USA) were applied as internal controls for nuclear and cytosolic proteins, respectively. The intensity of bands was quantified using AlphaView SA software.

### 2.15. Statistical Analysis

All experimental data are expressed as mean ± SEM values. Statistical analyses were performed by independent *t* test when data consisted of only two groups or by one-way analysis of variance (ANOVA) when more than two groups were compared. A *P* value less than 0.05 was considered as a statistically significant difference.

## 3. Results

### 3.1. SFN Ameliorated Pancreatic Damage in Cerulein-Induced AP in Mice

**Figure 1:**
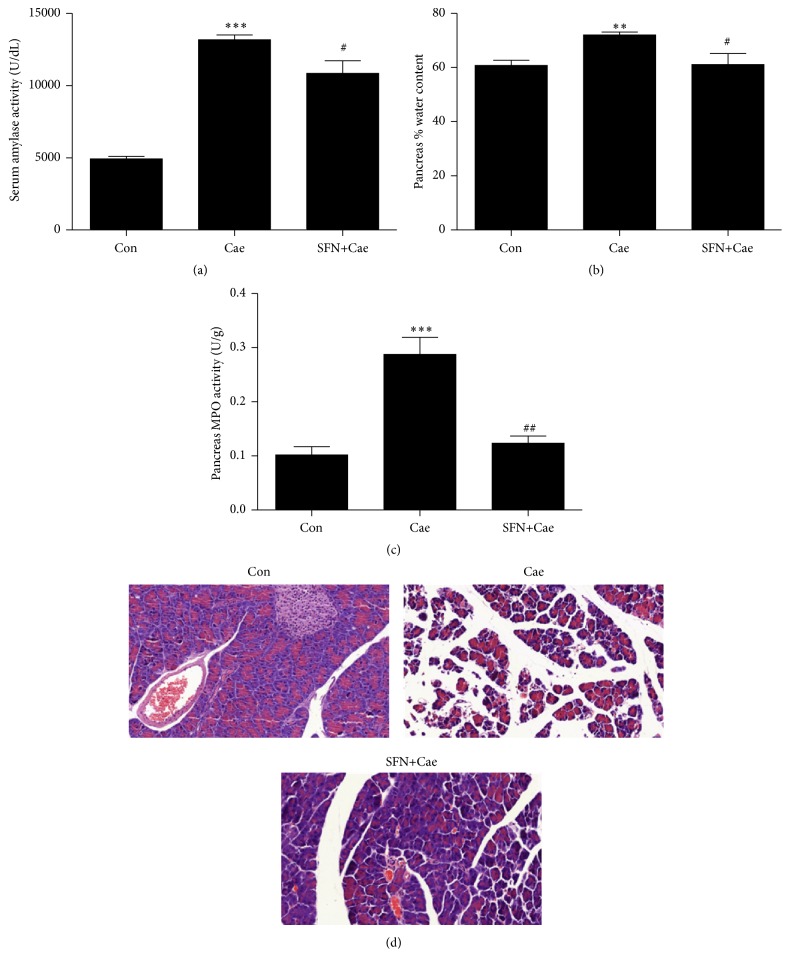
SFN administration protects against cerulein-induced AP in mice. The administration of SFN (5 mg/kg) in mice significantly attenuated the increase of serum amylase activity (a), pancreatic water content ((b) indicator of edema), pancreatic MPO activity ((c) indicator of leukocyte infiltration), and pancreatic acinar cell necrosis ((d) morphological examination), induced by cerulein. Values are expressed as mean ± SEM. ^*∗∗*^ *P* < 0.01 and ^*∗∗∗*^ *P* < 0.001 when cerulein-induced mice were compared with control mice. ^#^ *P* < 0.05 and ^##^ *P* < 0.01 when SFN-treated AP mice were compared to cerulein-induced mice.

As shown in Figures 1(a) and 1(b), pancreatic injury in mice with AP was confirmed by high levels of serum amylase and elevated pancreatic water content. Mice were administered SFN (5 mg/kg, i.p.) 3 days before AP induction by cerulein hyperstimulation. Prophylactic treatment with SFN attenuated pancreatic edema and serum amylase levels when compared with mice treated with cerulein alone. MPO is an enzyme principally located in neutrophil azurophilic granules and in monocyte lysosomes and is therefore used to measure the extent of such cell infiltration into tissues [25]. MPO activities of SFN-treated mice were reduced by 2.7-fold in comparison with cerulein-induced mice (Figure 1(c)). Histological examination of pancreas sections further confirmed the protection of SFN to cerulein-induced pancreatic damage (Figure 1(d)).

### 3.2. SFN Inhibited Oxidative Stress in the Pancreas during Cerulein-Induced AP

**Figure 2:**
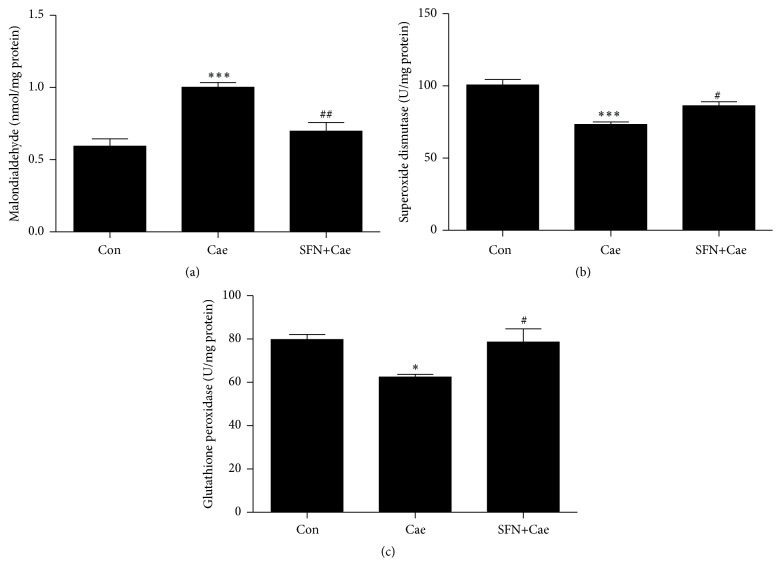
Effect of SFN administration on pancreatic levels of MDA, SOD, and GPx in mice with cerulein-induced AP. (a) Pancreatic MDA level (indicator of lipid peroxidation) after SFN treatment in mice with cerulein-induced AP was measured using the thiobarbituric acid (TBA) method. (b, c) Pancreatic levels of the SOD and GPx (antioxidant enzymes responsible for scavenging toxic metabolites generated by free radicals) after SFN treatment in mice with cerulein-induced AP were measured using commercial kits as described in Methods. Values are expressed as mean ± SEM. ^*∗*^ *P* < 0.05 and ^*∗∗∗*^ *P* < 0.001 when cerulein-induced mice were compared with control mice. ^#^ *P* < 0.05 and ^##^ *P* < 0.01 when SFN-treated AP mice were compared to cerulein-induced mice.

MDA, SOD, and GPx were analyzed in pancreas tissue homogenates as an indicator of lipid peroxidation and activity of antioxidant enzymes. Induction of AP with cerulein that resulted in elevation of MDA and pancreatic MDA in mice treated with SFN was reduced as compared with that in cerulein only-treated mice (Figure 2(a)). SOD and GPx are antioxidant enzymes responsible for scavenging toxic metabolites generated by free radicals. As shown in Figures 2(b) and 2(c), pancreatic SOD and GPx in the cerulein-induced group were significantly lower than those in the control group. Treatment with SFN caused elevated levels of the two antioxidant enzymes compared with cerulein-treated mice.

### 3.3. Nrf2 Activation Is Associated with SFN-Induced Inhibition of Oxidative Stress in Pancreatic Acinar Cells

**Figure 3:**
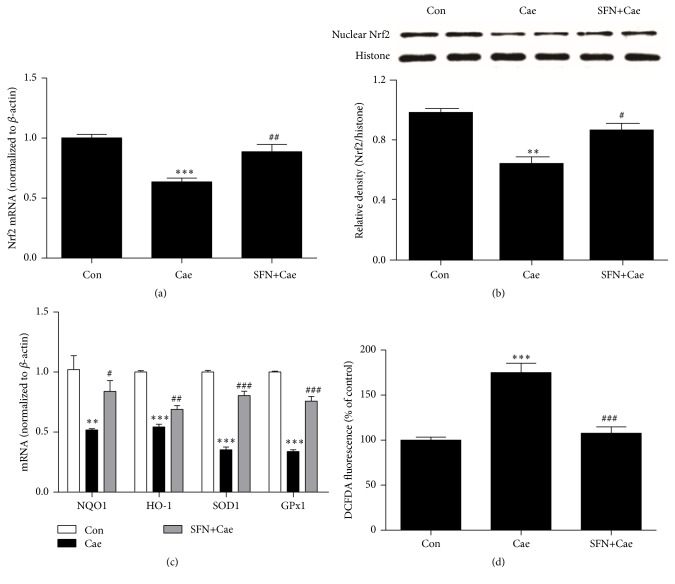
Effect of SFN administration on Nrf2 and Nrf2-regulated antioxidant enzyme expression and production of ROS during cerulein-induced AP. (a) The mRNA levels of Nrf2 after SFN treatment in cerulein-stimulated pancreatic acinar cells were measured by quantitative real-time PCR. (b) Expression of protein of nuclear Nrf2 by SFN in cerulein-treated pancreatic acinar cells was measured by western blot. (c) The mRNA expression of NQO1, HO-1, SOD1, and GPx1 after SFN treatment in cerulein-induced pancreatic acinar cells was measured by quantitative real-time PCR. (d) Lastly, the effect of SFN on the production of ROS in cerulein-treated pancreatic acinar cells was detected by fluorescence absorbance. Values are expressed as mean ± SEM. ^*∗∗*^ *P* < 0.01 and ^*∗∗∗*^ *P* < 0.001 when cerulein-induced group was compared with control group. ^#^ *P* < 0.05, ^##^ *P* < 0.01, and ^###^ *P* < 0.001 when SFN-treated group was compared to cerulein-induced group.

Nrf2 is a redox-sensitive transcription factor that becomes activated and translocates into the nucleus in response to oxidative stress [25]. To determine whether the Nrf2/ARE pathway contributed to the induction of antioxidant enzymes by SFN, we investigated the mRNA level and protein expression of Nrf2 in mouse primary pancreatic acinar cells. As shown in Figure 3(a), the mRNA expression level of Nrf2 in the cerulein-induced pancreatic acinar cells was lower than control cells. Nrf2 mRNA expression in cells treated with SFN was significantly elevated compared to cerulein-induced cells. In addition, SFN treatment increased nuclear translocation of Nrf2 compared with the cerulein-induced group (Figure 3(b)). To determine if Nrf2-regulated genes could be induced by SFN in cerulein-induced AP, mRNA expression of NQO1, HO-1, SOD1, and GPx1 was determined in cerulein-induced pancreatic acinar cells by Q-PCR. As shown in Figure 3(c), NQO1, HO-1, SOD1, and GPx1 mRNA expression in cells treated with SFN were all significantly higher than those of cerulein-induced group. Finally, we measured ROS levels to determine the effect of Nrf2 on oxidative stress (Figure 3(d)). Levels of ROS produced by cerulein-stimulated pancreatic acini cells were higher than those observed with unstimulated pancreatic acini. Notably, pretreatment of acini cells with 10 *μ*M SFN significantly decreased ROS levels in cerulein-stimulated acini cells.

### 3.4. SFN Inhibited NF-*κ*B Activation and Decreased Expression of Proinflammatory Cytokines in Cerulein-Treated Pancreatic Acinar Cells or Mice with AP

**Figure 4:**
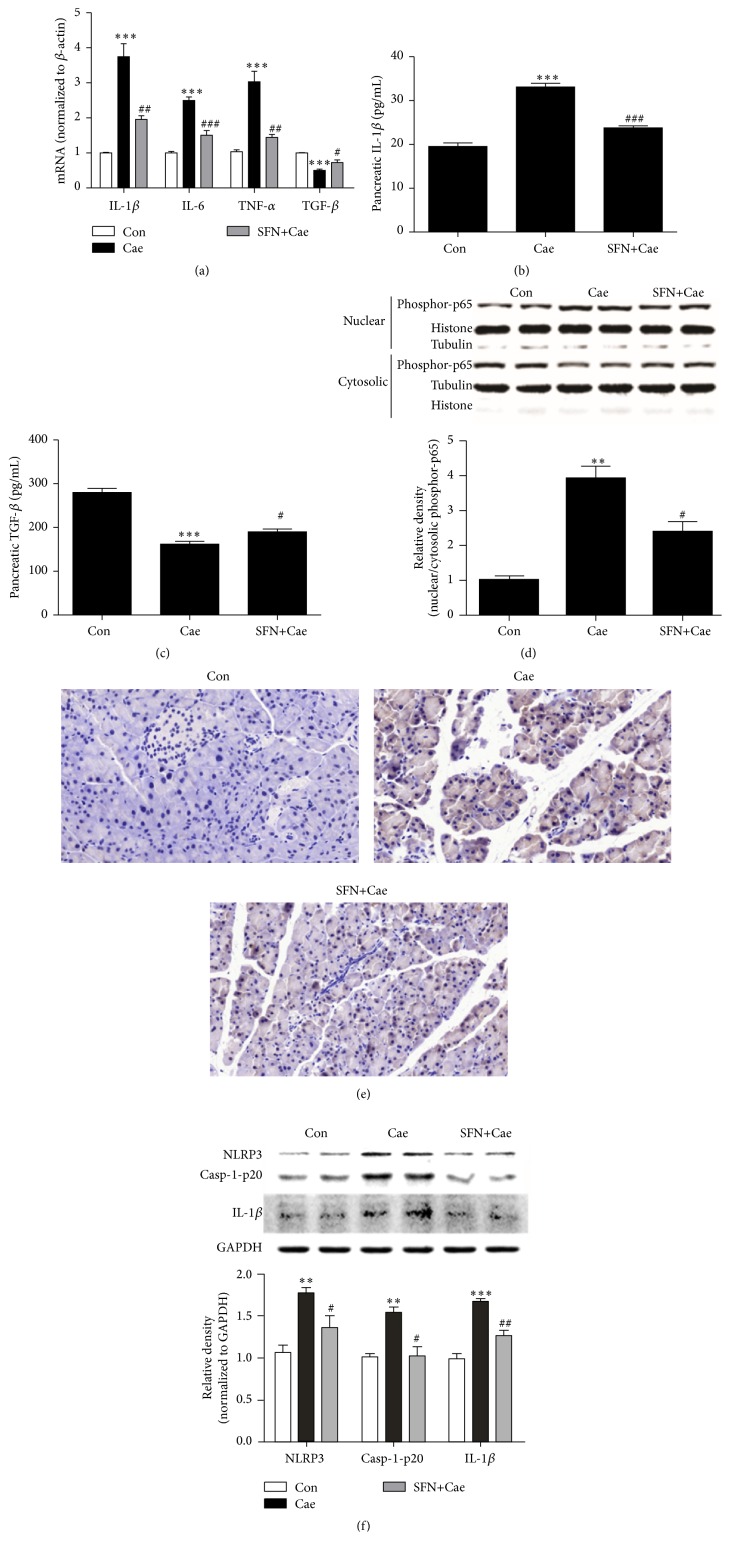
SFN modulates expression of inflammatory mediators, NF-*κ*B activity, and NLRP3 inflammasome during cerulein-induced AP. (a) The mRNA levels of pancreatic IL-1*β*, IL-6, TNF-*α*, and TGF-*β* after SFN treatment in cerulein-stimulated pancreatic acinar cells were measured by quantitative real-time PCR. (b, c) Correspondently, the protein levels of IL-1*β* and TGF-*β* were determined using quantitative ELISA kits. (d) The effect of SFN on phosphor-p65 protein expression in cerulein-treated pancreatic acinar cells was measured by western blot. (e) NF-*κ*B p65 protein expression in cerulein-induced mice was determined by immunohistochemistry. (f) The effect of SFN on cerulein-induced NLRP3 inflammasome activation was evaluated by detecting the protein levels of NLRP3, caspase-1-p20, and cleaved IL-1*β* in cerulein-induced acinar cells by western blot assay. Values are expressed as mean ± SEM. ^*∗∗*^ *P* < 0.01 and ^*∗∗∗*^ *P* < 0.001 when cerulein-induced group was compared with control group. ^#^ *P* < 0.05, ^##^ *P* < 0.01, and ^###^ *P* < 0.001 when SFN-treated group was compared to cerulein-induced group.

As the transcription factor NF-*κ*B is activated early in acinar cells during AP and regulates expression of multiple inflammation-related genes [30], we assessed the effects of SFN on NF-*κ*B activation and the expression of proinflammatory genes in cerulein-induced mouse primary pancreatic acinar cells. As shown in Figure 4(a), mRNA expression of proinflammatory mediators including tumor necrosis factor-alpha (TNF-*α*), interleukin- (IL-) 1*β*, and IL-6, upregulated in cerulein-induced mouse primary pancreatic acinar cells, was significantly reduced by SFN. ELISA analyses further confirmed that SFN inhibited proinflammatory cytokine (IL-1*β*) production and increased anti-inflammatory cytokine (TGF-*β*) production (Figures 4(b) and 4(c)) in mice with cerulein-induced AP. Subsequently, we assessed whether SFN had any effect on NF-*κ*B activation in cerulein-induced pancreatic acinar cells by western blot analysis. SFN treatment attenuated nuclear translocation of phosphor-p65 compared with cerulein-induced group (Figure 4(d)). Modulatory effect of SFN on p65 translocation was also observed by immunohistochemical staining and examination in mice. As shown in Figure 4(e), SFN administration attenuated enhanced p65 expression as well as its nuclear translocation induced by cerulein.

### 3.5. SFN Inhibited NLRP3 Inflammasome Activation in Cerulein-Stimulated Pancreatic Acinar Cells

As an important component of innate immune system, the NLRP3 inflammasome is involved in the development of inflammation during AP [19] and has been shown to regulate the maturation and release of IL-1*β* [31]. We have observed that augmented secretions of IL-1*β* in both AP mice and cerulein-stimulated pancreatic acinar cells were substantially suppressed by the treatment of SFN. Next, we assessed the effect of SFN on the activation of NLRP3 pathway in cerulein-induced pancreatic acinar cells. Cerulein stimulated the expression of NLRP3, casp-1-p20, and cleaved IL-1*β* in pancreatic acinar cells. SFN treatment markedly suppressed the expression of NLRP3 signaling proteins, suggesting its anti-inflammatory effects by inhibiting NLRP3 inflammasome activation (Figure 4(f)).

## 4. Discussion

The present study demonstrated that SFN protected cerulein-induced AP in mice by upregulating antioxidant enzymes including NQO1, HO-1, SOD, and GPx through Nrf2 activation. In addition, SFN attenuated inflammation in AP by inhibiting NLRP3 and NF-*κ*B inflammatory pathways. Thus, the dietary isothiocyanate compound SFN harbors positive modulatory effects on experimental AP by suppressing Nrf2-mediated oxidative stress as well as NLRP3 and NF-*κ*B inflammatory responses.

AP is characterized by premature activation of pancreatic enzymes leading to self-digestion, cellular inflammation, and damage. Accumulating evidence has demonstrated that oxidative stress is closely related and causative to the inflammatory responses and associated tissue damage [4–6, 9, 10]. Thus it is promising to seek a novel management strategy that focuses on antioxidant intervention, particularly during the early phase of AP, to prevent the development of SIRS and MODS. Interestingly, SFN as a highly effective phytochemical antioxidant in a number of pathophysiological studies has not been evaluated in AP [15–17].

SFN is an isothiocyanate derivative of its precursor glucoraphanin, a glucosinolate found in high amounts in broccoli and other* Brassica* species [32]. Kinetic studies of SFN in mice after consumption of SFN-enriched broccoli sprouts found that it was readily released, quickly absorbed, and distributed throughout the tissues [33, 34]. Following direct oral gavage of SFN in Nrf2 knockout and wild-type mice, its metabolites were detected in all tissues at 2 and 6 h after gavage. Furthermore, the relative abundance of each metabolite was not strikingly different between genders and genotypes [35]. These data suggest that SFN is bioavailable and may be an effective dietary antioxidant agent for several tissue sites. In previous studies, the treatment of SFN used as an antioxidant or a chemotherapy agent varies from hours* in vitro* to 1 week or several weeks* in vivo* [36–38]. Here a significant effect of SFN was observed with prophylactic treatment of 3 days prior to AP induction. Functionally, it has been suggested that SFN has protective roles in gastritis [15], acute colitis [16], and pulmonary damage [17], as well as for prevention of pancreatic cancer [20] and other cancers [39, 40]. In the present study, we extended the protective effects of SFN to cerulein-induced AP model as evidenced by attenuation in pancreatic edema and increases in serum amylase and pancreatic MPO levels and confirmed by histological examination of pancreas sections. Subsequently, we confirmed the antioxidant effects of SFN by measuring the levels of MDA, which is a reliable marker of lipid peroxidation and the endogenous antioxidant enzymes SOD and GPx in cerulein-induced AP. We found that, in mice with AP, SFN pretreatment caused a significant decrease in tissue MDA levels, accompanied with a significant increase in SOD and GPx activities. Indeed, SFN countered cerulein-induced increase in lipid peroxidation and decrease in antioxidative capacity.

SFN has been known as a potent activator of Nrf2, a major regulator of the constitutively expressed cytoprotective enzymes in various tissues and cell types [41, 42]. Nrf2 plays a critical role in maintaining cellular redox balance. Once activated, Nrf2 can escape from its cytosolic repressor Keap1-mediated proteasomal degradation. Stabilized Nrf2 translocates to the nucleus, where it binds to specific promoter sequences ARE and regulates the expression of a set of detoxifying and antioxidant enzymes [25].

NQO1, HO-1, SOD, and GPx are the most important antioxidant enzymes of Nrf2/ARE signaling pathway [43]. Activation of Nrf2/ARE and its downstream genes has been shown to protect against organ damage in ischemia/reperfusion injury [44, 45]. However, pathophysiological mechanisms of different conditions, severity, and progression point to differential effects on Nrf2 expression [46, 47]. In our study, Nrf2 expression in cerulein-treated mouse primary acinar cells was low compared with the untreated control, which was similar to what has been reported in cerulein-induced AP in rats [25]. SFN pretreatment to cerulein hyperstimulation induced Nrf2 activation and its nuclear translocation (Figure 3). These results are in agreement with previous reports in experimental autoimmune encephalomyelitis and in arsenic-induced pulmonary damage [17, 48]. In addition to Nrf2 activation, we observed the increase of NQO1, HO-1, SOD1, and GPx1 expression in cerulein-stimulated primary acini. Similar results have been reported in melatonin-induced antioxidant responses in mouse acinar cells [49]. Moreover, SFN has been reported to augment the cellular antioxidant defense capacity via the Nrf2/ARE signaling in pancreatic beta cells [50]. Hence, SFN is able to trigger the accumulation of Nrf2 in the nucleus to promote transcription of target genes including NQO1, HO-1, SOD1, and GPx1 in pancreatic acinar cells following cerulein-induced injury.

Suppression of inflammatory processes is key to the control of AP and its systemic complications. NF-*κ*B is a critical regulator of inflammation-related genes in the pancreas [51]. Activation of NF-*κ*B in acinar cells worsens pancreatitis severity in mice [30] and cerulein-induced pancreatitis is found to be attenuated in NF-*κ*B-deficient mice [52]. In addition, there has been a close relation between Nrf2 and NF-*κ*B activation. Nrf2-deficient mice display increased NF-*κ*B activation and disruption of Nrf2 causes enhanced NF-*κ*B activity and proinflammatory cytokine production [53, 54]. Here, we demonstrated that SFN decreased production of inflammatory mediators including TNF-*α*, IL-1*β*, and IL-6 and inhibited NF-*κ*B activation in cerulein-treatment acini or mice. These results are in line with previous reports in other models of inflammation [15, 16, 55].

Recently, NLRP3 inflammasome has emerged as an important player in the pathogenesis of AP [19]. Reduction of oxidative stress by the antioxidant hydrogen suppressed activation of NLRP3 inflammasome and maturation of IL-1*β* that have deepened current understanding of antioxidants in managing AP [12]. Our data revealed that NLRP3 inflammasome activation is associated with cerulein-induced inflammation in acini and suppressed by SFN. Earlier reports have suggested that SFN inhibited caspase-1 cleavage and IL-1*β* maturation for the NLRP1b, NLRP3, NAIP5/NLRC4, and AIM2 inflammasomes in immune cells [18]. This study, for the first time, has demonstrated that SFN blocked the NLRP3 inflammasome activation in cerulein-induced pancreatic acini.

**Figure 5:**
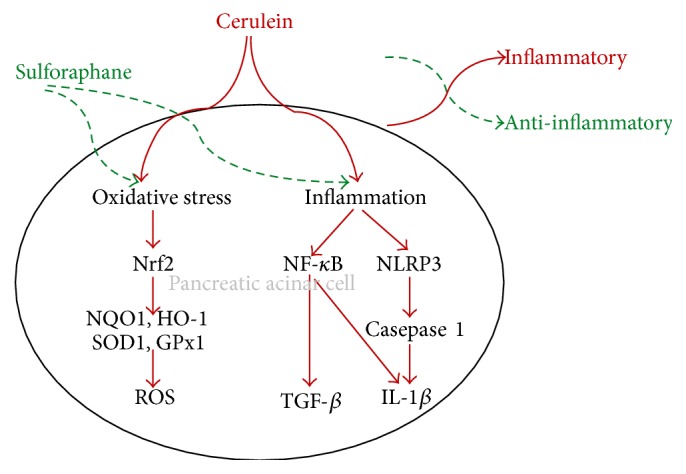
A schematic representation of the modulatory effects of SFN on cellular oxidative and inflammatory pathways during AP. Cerulein induces oxidative stress and inflammation in primary acinar cells or mice with AP. Prophylactic treatment of SFN restored steady-state pancreatic levels of MDA and redox enzymes including GPx and SOD, which were dysregulated towards enhanced oxidative state during AP. Further mechanistic investigation revealed that SFN enhanced the expression of Nrf2 in pancreatic acinar cells and Nrf2-regulated genes including NQO1, HO-1, SOD1, and GPx critical in the pathogenesis of AP. Moreover, SFN selectively suppressed cerulein-induced activation of NLRP3 inflammasome in pancreatic acinar cells, in parallel with decreased NF-*κ*B activity and reduced expressions of IL-1*β*, TNF-*α*, IL-6, and TGF-*β*. Together, SFN suppresses oxidative stress and inflammatory responses via Nrf2 and NLRP3 NF-*κ*B pathways, respectively, thereby protecting AP.

In conclusion, SFN attenuated pancreatic damage by exerting antioxidant defensive activities through the Nrf2 pathway and anti-inflammatory effects by inhibition of NLRP3 inflammasome and NF-*κ*B during AP (Figure 5).* Brassica*-derived isothiocyanate SFN may therefore represent a beneficial nutrient compound that can be used in managing AP.

## Figures and Tables

**Table 1:** Specific primers for qRT-PCR.

Target gene	Forward	Reverse
Nrf2	5′-TCCGCTGCCATCAGTCAGTC-3′	5′-ATTGTGCCTTCAGCGTGCTTC-3′
NQO1	5′-CAAGTTTGGCCTCTCTGTGG-3′	5′-AAGCTGCGTCTAACTATATGT-3′
HO-1	5′-AACAAGCAGAACCCAGTCTATGC-3′	5′-AGGTAGCGGGTATATGCGTGGGCC-3′
SOD1	5′-TGGGTTCCACGTCCATCAGTA-3′	5′-ACCGTCCTTTCCAGCAGTCA-3′
GPx1	5′-TCAGTTCGGACACCAGGAGAA-3′	5′-CTCACCATTCACTTCGCACTTC-3′
TNF-*α*	5′-AGGGTCTGGGCCATAGAACT-3′	5′-CCACCACGCTCTTCTGTCTAC-3′
IL-1*β*	5′-CTGAACTCAACTGTGAAATGC-3′	5′-TGATGTGCTGCTGCGAGA-3′
IL-6	5′-CTCTGCAAGAGACTTCCATCCAGT-3′	5′-GAAGTAGGGAAGGCCGTGG-3′
TGF-*β*	5′-GAACCAAGGAGACGGAATACAG-3′	5′-AACCCAGGTCCTTCCTAAAGTC-3′
*β*-actin	5′-GGCTGTATTCCCCTCCATCG-3′	5′-CCAGTTGGTAACAATGCCATGT-3′

## References

[1] Schick V., Scheiber J. A., Mooren F. C. (2014). Effect of magnesium supplementation and depletion on the onset and course of acute experimental pancreatitis. *Gut*.

[2] Zyromski N., Murr M. M. (2003). Evolving concepts in the pathophysiology of acute pancreatitis. *Surgery*.

[3] Pandol S. J., Saluja A. K., Imrie C. W., Banks P. A. (2007). Acute pancreatitis: bench to the bedside. *Gastroenterology*.

[4] Escobar J., Pereda J., Arduini A. (2009). Cross-talk between oxidative stress and pro-inflammatory cytokines in acute pancreatitis: a key role for protein phosphatases. *Current Pharmaceutical Design*.

[5] Schoenberg M. H., Buchler M., Gaspar M. (1990). Oxygen free radicals in acute pancreatitis of the rat. *Gut*.

[6] Tsai K., Wang S.-S., Chen T.-S. (1998). Oxidative stress: an important phenomenon with pathogenetic significance in the progression of acute pancreatitis. *Gut*.

[7] Talalay P., Dinkova-Kostova A. T., Holtzclaw W. D. (2003). Importance of phase 2 gene regulation in protection against electrophile and reactive oxygen toxicity and carcinogenesis. *Advances in Enzyme Regulation*.

[8] Fisher C. D., Augustine L. M., Maher J. M. (2007). Induction of drug-metabolizing enzymes by garlic and allyl sulfide compounds via activation of constitutive androstane receptor and nuclear factor E2-related factor 2. *Drug Metabolism and Disposition*.

[9] Dabrowski A., Konturek S. J., Konturek J. W., Gabryelewicz A. (1999). Role of oxidative stress in the pathogenesis of caerulein-induced acute pancreatitis. *European Journal of Pharmacology*.

[10] Hackert T., Werner J. (2011). Antioxidant therapy in acute pancreatitis: experimental and clinical evidence. *Antioxidants and Redox Signaling*.

[11] Niederau C., Niederau M., Borchard F. (1992). Effects of antioxidants and free radical scavengers in three different models of acute pancreatitis. *Pancreas*.

[12] Ren J.-D., Ma J., Hou J. (2014). Hydrogen-rich saline inhibits NLRP3 inflammasome activation and attenuates experimental acute pancreatitis in mice. *Mediators of Inflammation*.

[13] Liu H., Talalay P. (2013). Relevance of anti-inflammatory and antioxidant activities of exemestane and synergism with sulforaphane for disease prevention. *Proceedings of the National Academy of Sciences of the United States of America*.

[14] Ahn Y.-H., Hwang Y., Liu H. (2010). Electrophilic tuning of the chemoprotective natural product sulforaphane. *Proceedings of the National Academy of Sciences of the United States of America*.

[15] Yanaka A., Fahey J. W., Fukumoto A. (2009). Dietary sulforaphane-rich broccoli sprouts reduce colonization and attenuate gastritis in *Helicobacter pylori*–infected mice and humans. *Cancer Prevention Research*.

[16] Wagner A. E., Will O., Sturm C., Lipinski S., Rosenstiel P., Rimbach G. (2013). DSS-induced acute colitis in C57BL/6 mice is mitigated by sulforaphane pre-treatment. *Journal of Nutritional Biochemistry*.

[17] Zheng Y., Tao S., Lian F. (2012). Sulforaphane prevents pulmonary damage in response to inhaled arsenic by activating the Nrf2-defense response. *Toxicology and Applied Pharmacology*.

[18] Greaney A. J., Maier N. K., Leppla S. H., Moayeri M. (2016). Sulforaphane inhibits multiple inflammasomes through an nrf2-independent mechanism. *Journal of Leukocyte Biology*.

[19] Hoque R., Sohail M., Malik A. (2011). TLR9 and the NLRP3 inflammasome link acinar cell death with inflammation in acute pancreatitis. *Gastroenterology*.

[20] Kallifatidis G., Labsch S., Rausch V. (2011). Sulforaphane increases drug-mediated cytotoxicity toward cancer stem-like cells of pancreas and prostate. *Molecular Therapy*.

[21] Sharma A., Tao X., Gopal A. (2005). Protection against acute pancreatitis by activation of protease-activated receptor-2. *American Journal of Physiology-Gastrointestinal and Liver Physiology*.

[22] Bhatia M., Hegde A. (2007). Treatment with antileukinate, a CXCR2 chemokine receptor antagonist, protects mice against acute pancreatitis and associated lung injury. *Regulatory Peptides*.

[23] Singh V. P., Saluja A. K., Bhagat L. (2001). Phosphatidylinositol 3-kinase-dependent activation of trypsinogen modulates the severity of acute pancreatitis. *Journal of Clinical Investigation*.

[24] Niehaus W. G., Samuelsson B. (1968). Formation of malonaldehyde from phospholipid arachidonate during microsomal lipid peroxidation. *European Journal of Biochemistry*.

[25] Jung K. H., Hong S.-W., Zheng H.-M. (2010). Melatonin ameliorates cerulein-induced pancreatitis by the modulation of nuclear erythroid 2-related factor 2 and nuclear factor-kappaB in rats. *Journal of Pineal Research*.

[26] Gout J., Pommier R. M., Vincent D. F. (2013). Isolation and culture of mouse primary pancreatic acinar cells. *Journal of Visualized Experiments*.

[27] Tamizhselvi R., Koh Y.-H., Sun J., Zhang H., Bhatia M. (2010). Hydrogen sulfide induces ICAM-1 expression and neutrophil adhesion to caerulein-treated pancreatic acinar cells through NF-*κ*B and Src-family kinases pathway. *Experimental Cell Research*.

[28] Gibbs A., Schwartzman J., Deng V., Alumkal J. (2009). Sulforaphane destabilizes the androgen receptor in prostate cancer cells by inactivating histone deacetylase 6. *Proceedings of the National Academy of Sciences of the United States of America*.

[29] Zeng K.-W., Yu Q., Song F.-J. (2015). Deoxysappanone B, a homoisoflavone from the Chinese medicinal plant Caesalpinia sappan L., protects neurons from microglia-mediated inflammatory injuries via inhibition of I*κ*B kinase (IKK)-NF-*κ*B and p38/ERK MAPK pathways. *European Journal of Pharmacology*.

[30] Huang H., Liu Y., Daniluk J. (2013). Activation of nuclear factor-*κ*B in acinar cells increases the severity of pancreatitis in mice. *Gastroenterology*.

[31] Hornung V., Latz E. (2010). Critical functions of priming and lysosomal damage for NLRP3 activation. *European Journal of Immunology*.

[32] Paolini M., Perocco P., Canistro D. (2004). Induction of cytochrome P450, generation of oxidative stress and in vitro cell-transforming DNA-damaging activities by glucoraphanin, the bioprecursor of the chemopreventive agent sulforaphane found in broccoli. *Carcinogenesis*.

[33] Keum Y.-S., Oo Khor T., Lin W. (2009). Pharmacokinetics and pharmacodynamics of broccoli sprouts on the suppression of prostate cancer in transgenic adenocarcinoma of mouse prostate (TRAMP) Mice: implication of induction of Nrf2, HO-1 and apoptosis and the suppression of Akt-dependent kinase pathway. *Pharmaceutical Research*.

[34] Li Y., Zhang T., Li X., Zou P., Schwartz S. J., Sun D. (2013). Kinetics of sulforaphane in mice after consumption of sulforaphane-enriched broccoli sprout preparation. *Molecular Nutrition and Food Research*.

[35] Clarke J. D., Hsu A., Williams D. E. (2011). Metabolism and tissue distribution of sulforaphane in Nrf2 knockout and wild-type mice. *Pharmaceutical Research*.

[36] Bricker G. V., Riedl K. M., Ralston R. A., Tober K. L., Oberyszyn T. M., Schwartz S. J. (2014). Isothiocyanate metabolism, distribution, and interconversion in mice following consumption of thermally processed broccoli sprouts or purified sulforaphane. *Molecular Nutrition and Food Research*.

[37] Singh P., Sharma R., McElhanon K. (2015). Sulforaphane protects the heart from doxorubicin-induced toxicity. *Free Radical Biology and Medicine*.

[38] Wang L., Tian Z., Yang Q. (2015). Sulforaphane inhibits thyroid cancer cell growth and invasiveness through the reactive oxygen species-dependent pathway. *Oncotarget*.

[39] Matusheski N. V., Jeffery E. H. (2001). Comparison of the bioactivity of two glucoraphanin hydrolysis products found in broccoli, sulforaphane and sulforaphane nitrile. *Journal of Agricultural and Food Chemistry*.

[40] Xu C., Huang M.-T., Shen G. (2006). Inhibition of 7,12-Dimethylbenz(a)anthracene-induced skin tumorigenesis in C57BL/6 mice by sulforaphane is mediated by nuclear factor E2–related factor 2. *Cancer Research*.

[41] Elbling L., Weiss R.-M., Teufelhofer O. (2005). Green tea extract and (−)-epigallocatechin-3-gallate, the major tea catechin, exert oxidant but lack antioxidant activities. *The FASEB Journal*.

[42] Enomoto A., Itoh K., Nagayoshi E. (2001). High sensitivity of Nrf2 knockout mice to acetaminophen hepatotoxicity associated with decreased expression of ARE-regulated drug metabolizing enzymes and antioxidant genes. *Toxicological Sciences*.

[43] Chan K. H., Ng M. K. C., Stocker R. (2011). Haem oxygenase-I and cardiovascular disease: mechanisms and therapeutic potential. *Clinical Science*.

[44] Cheng L., Jin Z., Zhao R., Ren K., Deng C., Yu S. (2015). Resveratrol attenuates inflammation and oxidative stress induced by myocardial ischemia-reperfusion injury: role of Nrf2/ARE pathway. *International Journal of Clinical and Experimental Medicine*.

[45] Guo H., Li M.-J., Liu Q.-Q. (2014). Danhong injection attenuates ischemia/reperfusion-induced brain damage which is associating with Nrf2 levels in vivo and in vitro. *Neurochemical Research*.

[46] Wang Y.-Z., Zhang Y.-C., Cheng J.-S. (2014). Protective effects of BML-111 on cerulein-induced acute pancreatitis-associated lung injury via activation of Nrf2/ARE signaling pathway. *Inflammation*.

[47] Chen L., Wang L., Zhang X. (2012). The protection by Octreotide against experimental ischemic stroke: up-regulated transcription factor Nrf2, HO-1 and down-regulated NF-*κ*B expression. *Brain Research*.

[48] Li B., Cui W., Liu J. (2013). Sulforaphane ameliorates the development of experimental autoimmune encephalomyelitis by antagonizing oxidative stress and Th17-related inflammation in mice. *Experimental Neurology*.

[49] Santofimia-Castaño P., Clea Ruy D., Garcia-Sanchez L. (2015). Melatonin induces the expression of Nrf2-regulated antioxidant enzymes via PKC and Ca2+ influx activation in mouse pancreatic acinar cells. *Free Radical Biology and Medicine*.

[50] Song M.-Y., Kim E.-K., Moon W.-S. (2009). Sulforaphane protects against cytokine- and streptozotocin-induced *β*-cell damage by suppressing the NF-*κ*B pathway. *Toxicology and Applied Pharmacology*.

[51] Bhatia M., Brady M., Shokuhi S., Christmas S., Neoptolemos J. P., Slavin J. (2000). Inflammatory mediators in acute pancreatitis. *The Journal of Pathology*.

[52] Altavilla D., Famulari C., Passaniti M. (2003). Attenuated cerulein-induced pancreatitis in nuclear factor-*κ*B-deficient mice. *Laboratory Investigation*.

[53] Jin W., Wang H., Yan W. (2008). Disruption of Nrf2 enhances upregulation of nuclear factor-*κ*B activity, proinflammatory cytokines, and intercellular adhesion molecule-1 in the brain after traumatic brain injury. *Mediators of Inflammation*.

[54] Thimmulappa R. K., Lee H., Rangasamy T. (2006). Nrf2 is a critical regulator of the innate immune response and survival during experimental sepsis. *The Journal of Clinical Investigation*.

[55] Youn H. S., Kim Y. S., Park Z. Y. (2010). Sulforaphane suppresses oligomerization of TLR4 in a thiol-dependent manner. *The Journal of Immunology*.

